# Defining a Continuous Glucose Baseline to assess the impact of nutritional interventions

**DOI:** 10.3389/fnut.2023.1203899

**Published:** 2023-07-31

**Authors:** Célina Chkroun, Inez Trouwborst, Anna Cherta-Murillo, Lauren Owen, Christian Darimont, Andreas Rytz

**Affiliations:** ^1^Clinical Research Unit, Nestlé Research, Lausanne, Switzerland; ^2^Metabolic Health Department, Nestlé Institute of Health Sciences, Nestlé Research, Lausanne, Switzerland; ^3^Brain Health Department, Nestlé Institute of Health Sciences, Nestlé Research, Lausanne, Switzerland

**Keywords:** Continuous Glucose Monitoring, Continuous Glucose Baseline (24 h-CGB), nutritional intervention, basal glucose, fasting glucose

## Abstract

Accurate and robust estimation of individuals’ basal glucose level is a crucial measure in nutrition research but is typically estimated from one or more morning fasting samples. The use of Continuous Glucose Monitoring (CGM) devices presents an opportunity to define more robust basal glucose levels, which estimates can be generalized to any time of the day. However, to date, no standardized method has been delineated. The current paper seeks to define a reliable algorithm to characterize the individual’s basal glucose level over 24 h from CGM measurements. Data drawn from four nutritional intervention studies performed on adults free from chronic diseases were used to define that basal glucose levels were optimally estimated using the 40th percentile of the previous 24 h CGM data. This simple algorithm provides a Continuous Glucose Baseline over 24 h (24 h-CGB) that is an unbiased and highly correlated estimator (*r* = 0.86, *p*-value < 0.01) of standard fasting glucose. We conclude that 24-CGB can provide reliable basal glucose estimates across the day while being more robust to interference than standard fasting glucose, adaptable to evolving daily routines and providing useful reference values for free-living nutritional intervention research in non-diabetic individuals.

## 1. Introduction

The monitoring of blood glucose levels provides an important strategy in the prevention and management of Type 2 Diabetes (T2D) (1–4). High available carbohydrate meals are the main driver of acute increases in blood glucose levels (5). Clinical studies performed under a controlled setup are typically used to assess the impact of nutritional interventions and meals on Post-Prandial Glucose Response (PPGR) to guide patient or healthcare’s management decisions.

Traditionally, PPGRs are measured through blood sampling. Due to both the invasive nature of the technique and the analytical effort required to analyze blood samples, the number of gathered samples are kept minimal. Typical metrics used to quantify PPGRs are incremental Area Under the Curve (iAUC) or incremental maximal glucose values (iCmax). These incremental measures are calculated relative to a glucose baseline which generally is an individual-specific basal glucose level. Due to the limited number of samples that can be gathered, the basal glucose level is most of the time estimated from 1 or 2 samples collected after an overnight fast (6), prior to the nutritional intervention, and the PPGR is only assessed over 2 or 3 h through samples collected every 15–30 min. Hence, traditional studies are limited to assessing the short-term impact of nutritional interventions in the morning, after an overnight fast.

Although primarily developed to help T1D and T2D patients, Continuous Glucose Monitoring (CGM) devices are gaining popularity in healthy, normoglycemic and prediabetic people (7, 8). CGMs are now widely used in clinical research to replace blood sampling, reduce burden on study participants and investigational sites, and to provide high quantity of data going far beyond the 3 h post-prandial measures. CGMs further enable decentralized (9), free-living (10), and observational studies. The endpoints of such studies can be more diverse than only considering the 3 h post-prandial measures after an overnight fast. Typically, it is now possible to describe glucose excursions induced by the whole diet on the total 24 h glucose response, or by different eating occasions along the day. These descriptions can only be properly quantified if representative individual basal glucose levels were made available at any timepoint, typically before any eating occasion, or any other intervention, along the day.

The traditional methods to estimate basal glucose levels, developed when blood sampling was used and only limited amount of glucose data was available, present some limitations. First, only few closely spaced glucose measurements are considered in the computation. Hence, the estimate may only be valid in a short time window since basal glucose level may fluctuate during (11) and between (12) days. They may also be sensitive to interfering effects that are difficult to control, such as stress. Further, these methods relied on glucose measurements gathered after an overnight fast, under controlled conditions. For all these reasons, traditional methods may not be applicable to leverage the new analysis and endpoints enabled using CGM.

With CGM, more data are available, presenting new opportunities to estimate basal glucose levels, and overcome the limitations of the standard methods. In previous studies performed with CGM, different methods were used to estimate basal glucose levels, making comparisons between studies challenging. Specifically, calculations of the basal glucose differed in the number of glucose readings considered for the estimation, the time of the day that was used, or it relied on measurements gathered during previous days. Even with CGM, the basal glucose levels were generally estimated from few, closely spaced glucose readings gathered under controlled conditions, after an overnight fast; the considered data was for example reduced to one glucose reading (13), or to the average value estimated from all readings over 30 min (14), or over 60 min prior to nutritional interventions (10). In another study where no overnight fast was required (15), the single glucose reading gathered at 6 am was considered as estimate for basal glucose. In most of these studies (10, 13, 15), basal glucose was estimated for each test day, while in one of these studies (14), the estimate from the first day was considered for the analysis of all following days. These methods, besides being very diverse, all present similar limitations to the standard methods, while not taking profit of the richness of the whole CGM data.

In the present paper, we propose a new, continuously applicable method to estimate basal glucose levels considering glucose measurements collected over longer time period measured through CGM, that addresses the limitations of the current methods. This new method is developed with the aim to be generally applicable in all studies using CGM in non-diabetic individuals, and especially real-life studies. This method allows the quantification of the impact of any nutritional intervention along the day, with respect to a representative individual glycemic basal state and not relative to a non-representative transitory state.

Firstly, a method was developed and optimized using CGM data from multiple different studies where a standard method was used to estimate basal glucose. After that, the estimate computed using one method or the other were compared across all individuals, per study, or in some individuals. Finally, incremental metrics (2 h-iAUC and iCmax) were computed using both methods so that the impact of using the new method instead of the standard method was assessed on typical nutritional studies’ endpoints.

## 2. Methods

### 2.1. CGM data from 4 standardized nutritional intervention studies

The present study analyses CGM data of 67 subjects from four published nutritional intervention studies (9, 16, 17). In short, all four studies had a randomized cross-over design to compare the PPGRs of specific nutritional interventions, performed at breakfast after a minimum of 12 h of overnight fasting. All studies used the Abbott FreeStyle Libre sensor to automatically measure interstitial glucose every 15 min for several consecutive days (18). The main study characteristics regarding subjects, dietary interventions, and location are reported in Table 1.

**Table 1:** Overview of 4 studies including CGM data of 67 subjects.

	Study 1	Study 2*	Study 3	Study 4
Subjects (F:M)	16 (6:10)	27 (18:9)	14 (8:6)	10 (8:2)
Age [y]	31 (6)	30 (7)	49 (8)	32 (8)
BMI [kg/m^2^]	23.0 (1.6)	22.6 (1.9)	31.2 (2.8)	21.5 (1.9)
Fasting glucose [mmol/L]	4.8 (0.5)	5.0 (0.5)	5.4 (0.6)	4.7 (0.6)
Interventions/total days	4/9	3/8	6/9	4/5
Intervention type**	Ingredients	Supplements	Supplements	Ingredients
Set-up	Controlled	Controlled	Controlled	Decentralized

Study 1: (16), Study 2: (17), Study 3: (17), Study 4: (9). BMI, body mass index; F, female; M, male; y, years.

* 30 subjects were analyzed in this study, although complete CGM data were not available for three subjects.

** Ingredients: in the study, the glucose responses to different ingredients were assessed against the one of a high glycemic control ingredient; Supplements: the glucose response to the intake of supplements in addition to a standard meal was assessed against the one of the intakes of the standard meal alone.

The four studies were conducted on females and males free of chronic diseases. Study 3 included subjects at risk of developing T2D, aged 40–65 years with Body Mass Index (BMI) > 27 kg/m^2^. The three other studies included subjects aged 18–45 years with BMI 18.5–29.9 kg/m^2^. Other inclusion and exclusion criteria were similar between studies. The number and type of nutritional interventions was different between studies, with Study 1 and 4 comparing fully digestible carbohydrate ingredients and Study 2 and 3 assessing the effect of supplements on the PPGR of carbohydrate-rich meals. Interventions were performed at an investigational site with a controlled setup for Studies 1, 2 and 3 and at home, under close to real-life conditions, for Study 4.

Participants to the four studies signed an informed consent form as per local regulations and the study protocols were reviewed and approved by the Ethics Committee of Canton de Vaud and registered on clinicaltrials.gov as described in the respective publications. The current analyses were performed on anonymized CGM data, without any link to other personal data such as gender, age, or BMI, which makes that no health-related data could, without disproportional effort, be traced to a specific person. The objective of the performed analyses was the development of a statistical method to potentially improve the quality of the data analyses of the main endpoints of the four studies (i.e., iAUC and iCmax that both need the calculation of basal glucose value). These anonymized analyses were therefore aligned with the primary use of the data and did not require additional ethics approval.

### 2.2. Standard fasting glucose

In all four studies, the efficacy of the nutritional interventions was tested against a control food with high glycemic load with the objective to be able to significantly reduce the 2 h incremental glucose area under the curve (2 h-iAUC) and the incremental glucose peak (iCmax). Both endpoints require a basal glucose value against which the increment is calculated. The basal glucose in the studies was estimated using standard protocols recommending the use, after an overnight fast, of either the glucose level at time just before meal intake (T0), or the average of two values taken at T0 and 5 min before meal intake (T-5) (19).

### 2.3. 24 h-Continuous Glucose Baseline

The new method to estimate basal glucose was designed to be an unbiased estimator of the standard fasting glucose (difference between the estimate computed with the new and the standard method is null in average), while being generalizable to any time point, at any time of the day. The algorithm behind this new method estimated basal glucose level at a specific time as being a percentile of the glucose levels measured in a preceding time window. In the method development, the efficacy of different percentiles (every ten between the 20th and 80th percentile) and different time windows (6, 8, 10, 12, and 24 h) were assessed. Finally, the selected optimal method estimated basal glucose level as being the 40th percentile of the previous 24 h CGM data (24 h-Continuous Glucose Baseline, 24 h-CGB), because it provided a robust and unbiased estimate of the standard measure of basal glucose. As the algorithm uses the previous 24 h to compute the estimate, it always integrates the same information at any time of the day, namely a full-day routine, including both resting and active periods. It was specifically selected because it provided basal glucose estimates which can evolve with time, but which are not highly variable within individuals. Taking the 40th percentile over this time window allowed to calibrate the method to provide an estimate which is unbiased as compared to the standard estimate. Thanks to the large amount of datapoints considered for the estimation, the 24 h-CGB also allows to minimize the impact of interfering factors such as stress that are inherent to time T0 of many controlled interventions.

As 24 h of data are required to compute an estimate using the 24 h-CGB, it becomes available only 24 h after insertion of the sensor. The same is true in case of sensor loss with 24 h-CGB becoming again available only 24 h after starting a new sensor. In addition, 24 h-CGB can only be relevant if the amount of missing data over the last 24 h was minimal. This is ensured through the instruction to subjects to scan the sensor once at least every 8 h due to the limited storage capacity of the Free Style Libre sensor. Missing data, often related to sleep lasting longer than 8 h, were considered as such, without any imputation. 24 h-CGB was estimated at a given time-point only if missing data was less than 1/6 of the last 24 h data, corresponding to less than 4 h missing data which ensures that the daily routine could be properly captured. Standard fasting glucose and 24 h-CGB were expressed as mmol/L.

### 2.4. Comparison of 24 h-CGB vs. standard fasting glucose

**Figure 1:**
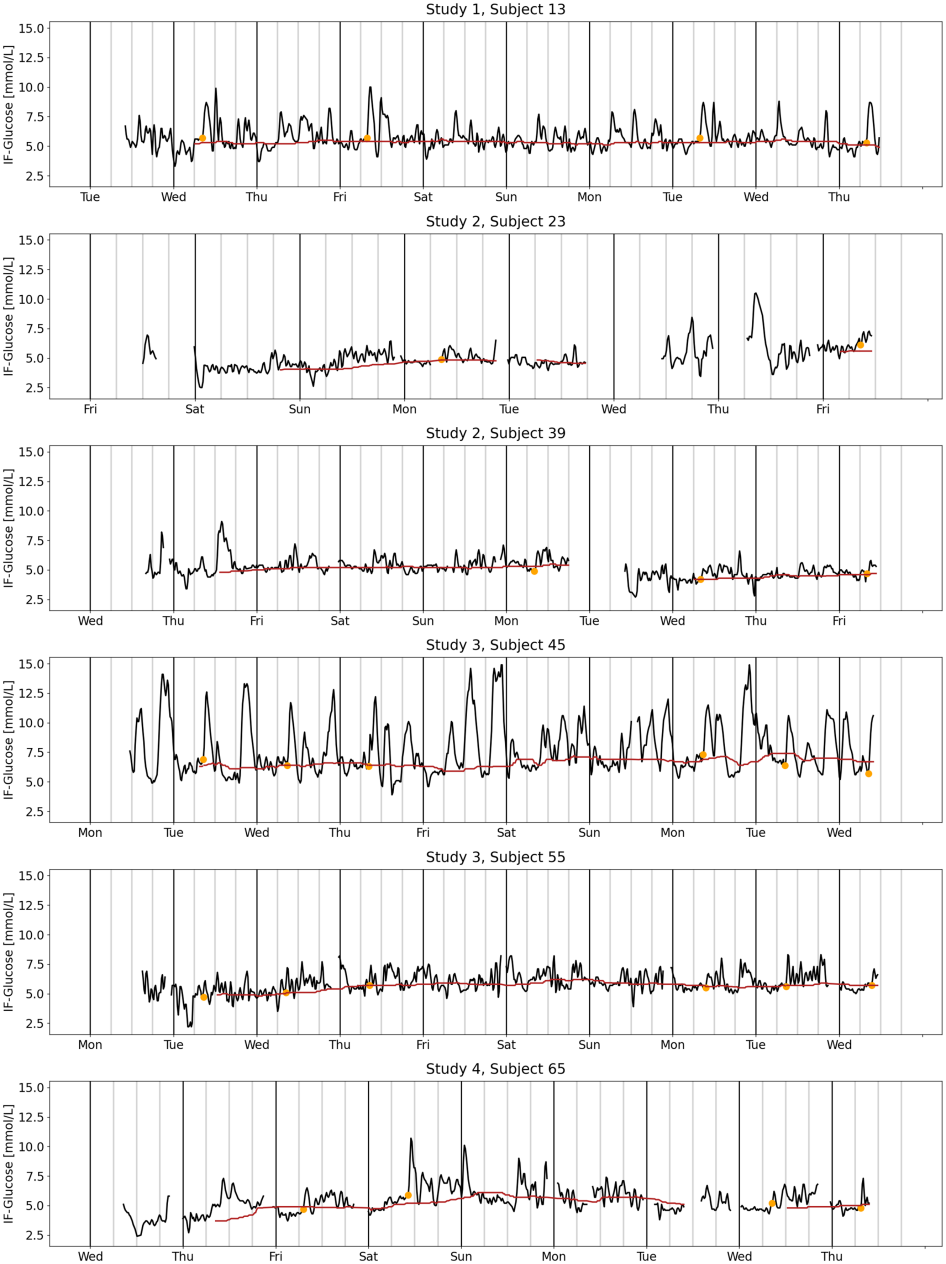
CGM signal (black line), 24 h-CGB (red line) and standard measures of fasting glucose (orange dot) for six representative subjects from four clinical studies. Study 1: (16), Study 2: (17), Study 3: (17), Study 4: (9). CGB, continuous glucose baseline; CGM, continuous glucose monitoring; IF, interstitial fluid.

**Figure 2:**
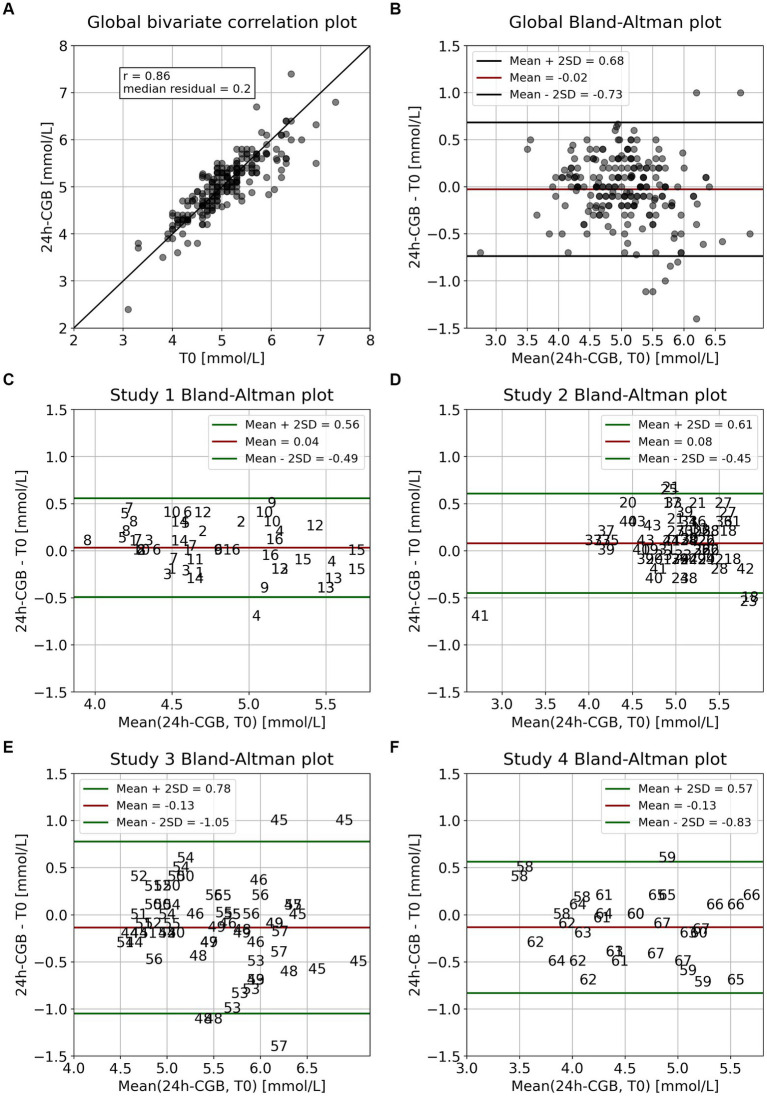
Comparison 24 h-CGB vs. T0 for interventions from four pooled studies. **(A)** Overall correlation plot, with identity line for visual guidance, **(B)** corresponding overall Bland–Altman plot, and **(C–F)** Bland–Altman plots by study with interventions identified by subject. Numbers represent participant’s identification numbers. Study 1: (16), Study 2: (17), Study 3: (17), Study 4: (9). CGB, continuous glucose baseline; SD, standard deviation; T0, standard fasting glucose.

**Figure 3:**
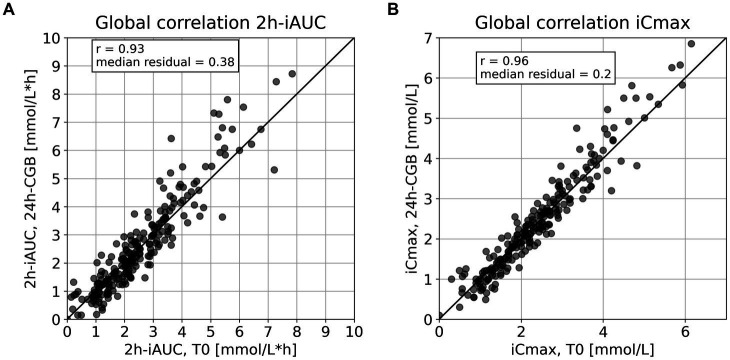
Comparison of incremental metrics using either the 24 h-CGB or T0 as baseline to quantify glucose responses for interventions from four pooled studies. **(A)** Correlation plot of 2 h-iAUC computed using T0 vs. 24 h-CGB, with identity line for visual guidance, **(B)** correlation plot of iCmax computed using T0 vs. 24 h-CGB, with identity line for visual guidance. CGB, continuous glucose baseline; T0, standard fasting glucose; iAUC, incremental Area Under the Curve; iCmax, incremental glucose peak.

CGM signals of six representative subjects are visualized together with the 24 h-CGB and the pre-meal fasting glucose values measured at T0 (Figure 1). This visualization is used to describe differences between continuous 24 h-CGB and fasting glucose considering the full CGM signals. A quantitative comparison of 24 h-CGB and fasting glucose is given using a bivariate correlation plot and Bland–Altman plots (20) (Figure 2). 2 h-iAUC and iCmax of the nutritional interventions of the original studies computed using either standard fasting glucose or the 24 h-CGB estimate as baseline are also compared using bivariate correlation plots (Figure 3). Finally, a comparison of the two approaches is performed in terms of sample size required to detect a reduction of 30% for 2 h-iAUC and of 25% for iCmax, compared to the high-glycemic control of each study to assess if using the 24 h-CGB method to compute incremental values implies that a greater sample size is needed to obtain the same study power as when using standard fasting values. These sample size calculations are performed separately for the four studies, using a conservative approach relying on a two-sample *t*-test, with *α* = 5% one-sided, and power of 80% (Table 2).

**Table 2:** Sample size* (N) required to detect decreases of 30% for 2 h-iAUC, and of 25% for iCmax, versus high-glycemic controls, for which the mean is tabulated together with the pooled between-subject standard deviation (SD).

	T0 method		24 h-CGB method	
	Mean (SD)	*N*	Mean (SD)	*N*
2 h-iAUC [mmol/l*h]				
Study 1	3.11 (1.15)	10	3.12 (1.35)	13
Study 2	2.80 (1.01)	9	2.92 (1.10)	10
Study 3	4.23 (1.81)	13	4.06 (2.17)	20
Study 4	2.70 (0.95)	9	3.49 (1.2)	9
iCmax [mmol/l]				
Study 1	3.56 (0.84)	6	3.50 (0.95)	8
Study 2	2.44 (0.73)	9	2.51 (0.77)	10
Study 3	3.79 (1.26)	11	3.49 (1.51)	19
Study 4	3.49 (0.85)	6	3.81 (0.94)	6

Incremental values were considered relative to the basal glucose level estimated at *t* = 0 min of interventions either through T0 or 24 h-CGB method.

Study 1: (16), Study 2: (17), Study 3: (17), Study 4: (9). CGB, continuous glucose baseline; N, sample size; SD; standard deviation; T0, standard fasting glucose; iAUC, incremental area under the curve; iCmax, incremental glucose peak.

* The sample sizes were computed from a one-sided two-sample *t*-test, with a significance level of 5%, and a power of 80%.

## 3. Results

### 3.1. Description of CGM, 24 h-CGB and standard fasting glucose

The CGM traces of the 67 studied subjects feature 5–9 days and show a large diversity in terms of number of daily peaks, height of peaks and glucose variability. This is exemplified by the traces of six representative subjects (Figure 1). Subject 45 shows larger glucose variability as compared to the five other subjects. The CGM data is almost complete for subjects 13, 45 and 55 revealing a high compliance with the requirement to scan at least once every 8 h. Subject 65 was compliant for the first days and less during the last, while subjects 23 and 39 needed to replace a lost sensor. These losses of sensors illustrate that glucose measures are sensor dependent, with the second sensor being positively biased compared to the first for subject 23 and negatively biased for subject 39. This sensor dependency underlines the importance of running sensor-dependent analyses to characterize the impact of nutritional interventions on glucose metabolism, by using endpoints that are incremental relative to a basal value, such as 2 h-iAUC or iCmax.

The 24 h-CGB captures the diversity of CGM-routines. Some subjects, such as 13 and 39, exhibit nearly stable 24 h-CGB, which could be likely due to constant daily routines over the full week. For other subjects, such as 55 and 65, the 24 h-CGB was higher on weekends, suggesting daily routines that vary over the week. Particularly, for subject 55 which seemingly may have variable daily routines, when comparing the 24 h-CGB with the fasting glucose values at T0, they match for all interventions. This suggests that even when there was a change in daily routine, the 24 h-CGB estimated fasting glucose similarly to T0 (when this was available). On the other hand, for subject 13, who has a constant daily routine, 24 h-CGB was below the fasting glucose value at time T0 for all interventions. This systematic bias may be related to a raise of glucose 1 h before T0. Subjects with patterns comparable to the one of subject 13 were observed in all studies except for Study 4 which was decentralized, suggesting that this bias may have been related to the stress induced by the timely venue to the investigational site. In such cases, 24 h-CGB appears to be more representative of the true basal glucose than the standard fasting glucose.

### 3.2. Comparison 24 h-CGB vs. standard fasting glucose

Standard fasting glucose at time T0 was available for 219 interventions and ranged between 3.1 and 7.3 mmol/L (mean = 5.06, SD = 0.68). The corresponding estimates of the 24 h-CGB ranged between 2.4 and 7.4 mmol/L (mean = 5.04, SD = 0.64). When comparing T0 vs. 24 h-CGB, the correlation is r = 0.86 (*p*-value<0.01) and the median absolute difference (MAD) = 0.20 mmol/L (Figure 2A). The corresponding Bland–Altman plot reveals that 24 h-CGB is an overall unbiased estimator of the standard fasting glucose measure (bias = −0.02 mmol/L) with a 95% confidence interval (95% CI) being −0.73–0.68 mmol/L (Figure 2B). The study specific Bland–Altman plots reveal that 24 h-CGB show minimal bias for all study setups, with bias = +0.04 mmol/L for Study 1, +0.08 mmol/L for Study 2, −0.13 mmol/L for Study 3 and 4. The 95% CI is smallest for Study 1 (±0.52 mmol/L), Study 2 (±0.53 mmol/L) and, slightly larger for Study 4 (±0.70 mmol/L) and larger for Study 3 (±0.92 mmol/L). This latter study furthermore features outliers such as subjects 45, 48, and 57 for which the difference is more than 1 mmol/L for some interventions. These subjects reveal the highest glucose variability, which might be related to the inclusion of older and more overweight subjects in this study than in the other studies.

### 3.3. Comparison 2 h-iAUC, iCmax computed using either standard fasting glucose or 24 h-CGB as baseline

Across all interventions where 24 h-CGB is defined, 2 h-iAUC computed using T0 and 24 h-CGB are correlated with *r* = 0.93 (*p*-value<0.01) and have a MAD of 0.38 mmol/L*h (Figure 3A). Computing iCmax with both techniques leads to a higher correlation of *r* = 0.96 (*p*-value < 0.01) and presents a MAD of 0.20 mmol/L. Both these correlations are higher than the correlation between standard fasting glucose and 24 h-CGB.

### 3.4. Comparison of required sample sizes when using 24 h-CGB vs. standard fasting glucose

Using standard fasting glucose, the sample size required to detect a 30% reduction in 2 h-iAUC vs. the highly glycemic control is 9–10 subjects for the three studies featuring healthy subjects aged 18–45 years, with BMI 18.5–29.9 kg/m^2^ (Table 2). This is in-line with standard recommendations of *N* = 10 (19) or *N* = 12 (21). The required sample size is slightly higher, namely *N* = 13, for Study 3 which features subjects aged 40–65 years with BMI > 27 kg/m^2^.

24 h-CGB appears to give a more robust estimate for Study 4, with minimally controlled conditions that are close to real-life, for which the sample size is kept constant at *N* = 9. The sample size slightly increases from *N* = 9 to 10 for Study 2 and from *N* = 10 to 13 for Study 1. This shows the ability of 24 h-CGB to perform well also in fully controlled setups. The sample size increase from *N* = 13 to 20 for Study 3 which shows that 24-CGB might be less appropriate for subjects with high glucose variability.

The sample sizes required to identify interventions that would decrease iCmax by 25% vs. a high glycemic control intervention are overall lower than those to detect a 30% reduction in the 2 h-iAUC. The ratios of sample sizes using T0 vs. 24 h-CGB method to compute iCmax are similar to those obtained for 2 h-iAUC (Table 2).

## 4. Discussion

The presented results show that the newly developed 24 h-CGB can generally replace the standard fasting glucose as estimate of basal glucose in controlled nutritional intervention studies performed in non-diabetic individuals. When considering typical endpoints characterizing PPGRs, it appears that the two estimates of basal glucose led to comparable estimates for both iCmax and iAUC, with an even higher correlation for iCmax (*r* = 0.96) than iAUC (*r* = 0.93). The sample sizes required to detect a relevant change vs. a highly glycemic control product are comparable when using 24 h-CGB or standard fasting glucose as baseline. The sample size was identical in case of the decentralized study, showing that the 24 h-CGB was particularly relevant in studies with reduced control, such as decentralized intervention studies or free-living observational studies. For two other studies performed under a more controlled set-up, the sample sizes required when using 24 h-CGB were minimally higher. This shows that 24 h-CGB is applicable in highly controlled nutritional studies without noticeable reduction in power, while being possibly extended to studies where there was less control, typically no strict overnight fasting. Finally, the samples sizes increased for the study including subjects at risk of developing T2D, and for which CGM profiles with greater variability were observed. Hence, the 24 h-CGB might be less powerful for controlled intervention studies including subjects with high glucose variability.

The 24 h-CGB provides a robust and continuously defined estimate for basal glucose level using CGM data, addressing the limitations of the current methods. Because of the continuous nature of this new estimate and its applicability at any time, its use can go far beyond traditional nutritional intervention studies, to leverage studies performed using CGM including new types of endpoints. Indeed, the 24 h-CGB estimates basal glucose levels, which can be used as reference glucose levels, against which glucose values from any time can be compared to. For example, short- or long-term glucose responses to nutritional interventions can be quantified during the day considering incremental values from basal glucose levels. The deviation of glucose levels with respect to basal glucose levels during the night following a nutritional intervention can also be evaluated and linked to sleep quality or other measures gathered with digital health sensors. The main driver to develop this method was its applicability to assess the impact of nutritional interventions and eating occasions along the day. Nevertheless, its use can be extended to analyze the impact of physical or pharmacological interventions on glucose levels.

During the design of the method, different parameters and algorithms were assessed. In terms of percentiles, the 20th, 60th, and 80th percentile led to greater biases compared to the measured fasting glucose baseline, thus being less appropriate. In terms of time windows, windows shorter than 24 h resulted in a large variation during the day, due to the relatively large impact of specific events in the preceding hours, such as meal intake, research study visit-induced stress, or sleep. Thus, including 24 h of glucose values to compute the 24 h-CGB estimate prevents it from being largely affected by recent events. In terms of algorithm, another method estimating basal glucose from glucose levels gathered during night sleep was evaluated. However, it was challenging to establish an accurate sleeping window for subjects without other digital health sensors or direct self-report of start and finish sleeping times. Additionally, during sleep, glucose levels remain stable or decrease minimally, which is not representative of 24 h-glycemic profiles. For instance, van Cauter et al. (22) reported that daytime fast resulted in a decrease of blood glucose from 5.3 to 4.2 mmol/L in healthy subjects, whereas nocturnal glycemia oscillated around 5.0 mg/dL.

This study has some strengths such as the calibration of the 24 h-CGB using data from a decentralized study, potentially capturing circumstances that extenuate blood glucose response such as stress or illness. Furthermore, we showed that the 24 h-CGB handles potential shifts in glucose levels due to CGM sensor-replacement, which is a common issue in CGMs (23). Compared to an isolated/discrete fasting glucose, which may be affected by previous meal intakes, physical activity (24–26), the 24 h-CGB appeared to be less subject to interfering effects (e.g., stress of attending a clinical visit). Furthermore, 24 h-CGB accounted for inter-day glucose variation and adapted to changes in daily routines.

However, this study also has some limitations. Firstly, considering the small amount of data available, the 24 h-CGB method was calibrated and assessed on the same dataset. However, as it performed almost equally well for four studies with different characteristics (in terms of subjects, interventions and/or setups), it shows that it has big potential to be generalizable. Secondly, the 24 h-CGB was calibrated using fasting glucose baselines in the morning, whereby the use of the 24 h-CGB to quantify glucose responses to intervention in the afternoon or evening should be performed with caution, as glucose homeostasis may be different depending on the time of day (11). Finally, the data that was used to calculate the 24 h-CGB derived mainly from healthy adults, potentially limiting its applicability to persons within a different age group or to categories of persons with other metabolic parameters whose glucose control upon the same nutritional challenge may differ (27, 28), or to other type of cohorts characterized by particular sleeping and eating patterns resulting in different daily glucose profiles such as shift-workers and infants (29). As discussed above, we already saw that the use of the 24 h-CGB resulted in less robust estimate for Study 3 including subjects at risk of T2D, which were observed to feature higher glucose variability.

In conclusion, we developed a new and robust method to continuously estimate basal glucose levels of individuals using CGM data. The method was based on daily glucose profiles and allowed to eliminate the acute impact of pre-intervention events on glucose responses and CGM-sensor failure-derived glucose shifts. The 24 h-CGB may be applicable in many studies with diverse endpoints enabled using CGMs, where standard estimation techniques were not applicable or limited. Mainly, it allowed for the quantification of short- or long-term glucose responses at any time, as well as providing reference glucose values to which measured glucose levels can be assessed. In future studies, the method should be assessed and optimized in other cohorts such as infants, prediabetic, obese, non-white Europeans and shift-workers.

## Data availability statement

The data analyzed in this study is subject to the following licenses/restrictions: the data used in this method development belong to 4 studies that have been published elsewhere and that can be requested from their authors. Requests to access these datasets should be directed to CC, Celina.Chkroun@rd.nestle.com.

## Author contributions

CC and AR: formal analysis and data curation. CC, IT, AC-M, and AR: original draft preparation. CC: visualization. CD: supervision. CC, IT, AC-M, LO, CD, and AR: conceptualization and writing—review and editing. All authors have read and agreed to the published version of the manuscript.

## Conflict of interest

CC, IT, AC-M, LO, CD, and AR are employed by Société des Produits Nestlé.

## Publisher’s note

All claims expressed in this article are solely those of the authors and do not necessarily represent those of their affiliated organizations, or those of the publisher, the editors and the reviewers. Any product that may be evaluated in this article, or claim that may be made by its manufacturer, is not guaranteed or endorsed by the publisher.
